# Cuticle and skin cell walls have common and unique roles in grape berry splitting

**DOI:** 10.1038/s41438-021-00602-2

**Published:** 2021-08-01

**Authors:** Ben-Min Chang, Markus Keller

**Affiliations:** Department of Horticulture, Irrigated Agriculture Research and Extension Center, Washington State University, Prosser, WA USA

**Keywords:** Plant physiology, Cell wall

## Abstract

The skin protects a fruit from environmental stresses and supports the fruit’s structure. Failure of the skin leads to fruit splitting and may compromise commercial production for fruit growers. The mechanical properties of the cuticle and skin cell walls might influence the splitting susceptibility of fleshy fruits. Thin shell theory and fracture mechanics were utilized in this study to target the potential factors contributing to splitting susceptibility. The study analyzed the structure of the cuticle and epidermis in ripening grape berries and examined the temporal dynamics of berry splitting. Cuticular waxes were partially removed, and skin cell walls were manipulated using wall stiffening and loosening solutions that altered reactions involving hydrogen peroxide. A more than twofold difference in cuticle thickness among grape cultivars did not account for their differences in splitting resistance. However, while removing predominantly epicuticular wax did not alter the berries’ splitting resistance, their surface appearance and increasing yield strength following partial wax removal support the notion that cuticular waxes contribute to berry mechanical properties. Immersing berries in H_2_O_2_-based cell wall loosening solutions increased the splitting probability and accelerated berry splitting, whereas cell wall stiffening solutions decreased the splitting probability and delayed berry splitting. These results showed that both cuticle and skin cell walls contribute to the mechanical properties of grape berries and to their splitting resistance. The results also suggest that the two current explanations for fruit splitting, the critical turgor model and the zipper model, should be viewed as complementary rather than incompatible.

## Introduction

The skin (exocarp) is the thin outer layer of a fleshy fruit, and it is made up of the composite cuticle, the epidermis, and the hypodermis^1,2^. The cuticle is crucial to aerial plant parts because its hydrophobic matrix minimizes material exchanges between plant cells and the environment. The matrix is a network of cutin imbued with waxes. In fruit, this extracellular membrane provides protection from biotic or abiotic stresses and helps the fruit to ripen successfully to disperse the seeds for reproducing the next generation^3^. Cuticles are secreted by epidermal cells and are considered an extension of those cells’ outer cell walls^3,4^. In addition to its protective role, the skin also limits fruit growth^5^. Continued expansive growth, however, may jeopardize the integrity of the cuticle and/or skin^6^. Existing microcracks in the cuticle can extend to become cracks in the fruit skin. Cracking poses risks for yield and quality in fruit production. Moreover, a crack can spread into the fruit flesh (mesocarp); this extension of cracking is termed fruit splitting^7^. Grape (*Vitis spp*.) berries, like other fleshy fruits, are susceptible to splitting. The driving forces for berry splitting include excessive internal pressure^8–10^, excessive surface-water absorption^11^, or physical damage, e.g. by insects or birds^12^.

Fracture mechanics in combination with shell theory may be a useful tool to explain the behavior during fruit splitting. In brief, fracture toughness^13^ is the energy required to create a new surface or break chemical bonding in a material. The fracture toughness, the applied stress (*σ*), and the crack size are the major factors that determine whether an existing crack will extend; such extension is termed fracture propagation. Before fracture propagation, *σ* causes strain *ε* = (L′−L)/L, where L and L′ are the dimensions before and after deformation due to *σ*, respectively. The sensitivity of *ε* to *σ* is defined as Young’s modulus or elastic modulus *E* = *σ*/*ε*, which is a measure of a material’s resistance to elastic deformation^14^. Tensile stress (*σ*_*h*_), which is of interest here, works on a plane in opposing directions and orients tangentially on the surface of a sphere. In a sphere with a thin (<10% of sphere radius) outer shell composed of a homogenous material, *σ*_*h*_ may be quantified by applying thin shell theory:

1 $$\sigma _h = \frac{{P_ir}}{{2t_s}}$$

where *P*_*i*_ is the internal pressure against the shell, *r* is the radius of the pressurized sphere, and *t*_*s*_ is the shell thickness^15^. Although a fruit skin is not a homogeneous material, shell theory has been successfully applied to fruits as a convenient simplification^10,16^. For instance, recent work found that immature, green-hard grape berries behaved like thick-shell spheres but changed to pressurized thin-shell spheres during berry softening at the onset of ripening and suddenly became susceptible to splitting^11^. Moreover, pressurizing the root system of grapevines led to reversible increases in *r* of immature berries but resulted in the splitting of ripening berries^8,17^, and restricting berry transpiration, which serves as a mechanism to relieve *P*_*i*_, increased the splitting frequency sixfold compared with the control in the absence of free water on the berry surface^9^.

While berry skin cell turgor is similar to or higher than flesh cell turgor^18,19^, skin cells are smaller and have thicker cell walls than flesh cells^1^. Therefore, skin cell walls have higher *E* than flesh cell walls to resist the cell turgor and restrict cell expansion during ripening^5,8,10^. In other words, unlike in immature (i.e., hard) berries, the comparatively low *E* of flesh cells in ripening (i.e., soft) berries prevents the flesh from dissipating *P*_*i*_ which is therefore transmitted to the skin^10^. However, defining stress-bearing and nonstress-bearing structures in a real fruit is challenging, and the stress-bearing structure might not be homogeneous material. Previous studies variously proposed the cuticle in tomato^20^, the epidermis, and hypodermis in sweet cherry^21^, and the cuticle and skin cell walls in apple^2^ as the major structures resisting fruit splitting. Nevertheless, the overall tension (*T*) in the skin can be estimated by Eq. 2 when *P*_*i*_ and *r* can be measured and the distribution of stress within the stress-bearing structure is ignored^22^.

2 $$T = \frac{{P_ir}}{2}$$

The framework of fracture mechanics and shell theory also implies that removal of cuticular wax and manipulation of cell wall stiffness may be used as tools to alter the shell strength. Wax embedded in the cutin matrix functions as a filler to stiffen the cuticle, and wax removal decreased the maximum *σ*_*h*_ and *E* in cuticles isolated from fruit skins^23^. In addition, hydroxyl radicals and other reactive oxygen species (ROS), which are involved in cell wall loosening and fruit softening^14,24^, might be useful to alter skin strength. Reactive oxygen species strengthen cell walls by cross-linking between polysaccharides, proteins, and phenolics^25,26^. Such cross-linkage occurs via oxidation, whereby hydrogen peroxide (H_2_O_2_) acts as an oxidant and wall-bound peroxidase (POX) acts as a catalyst^27,28^. In grape berries, POX is mostly localized in the skin, and skin cell walls have higher POX activity than flesh cell walls^29^. In addition, skin cell wall polysaccharides are dominated by hemicellulose with arabinose side chains^30^. The arabinans protect pectin polymers from attack by endo-polygalacturonase or pectin lyase. Indeed, the arabinoxylan in skin cell walls might provide active sites for phenolic cross-linkages^31^. It is possible that the configuration of cell wall polymers is altered by different biochemical pathways at the same time. Given that genes encoding diamine oxidase and polyamine oxidase are upregulated during grape ripening^32^, exogenous spermidine (Spd) might be the fuel to produce H_2_O_2_ and trigger skin cell wall stiffening^33^.

The aim of the present study was to evaluate the roles of the cuticle and skin cell walls in grape berry splitting. Building on earlier results that found that berry softening at the onset of ripening was associated with a rapid change in rheological properties and loss of splitting resistance^10^, we hypothesized that the cuticle and skin cell walls together form the thin shell of a spherical berry. The specific objectives of this study were to (1) determine the role of the cuticle and/or skin cell walls in bearing tensile stress, and (2) evaluate the effects of ROS on the probability of berry splitting.

## Results

**Fig. 1:**
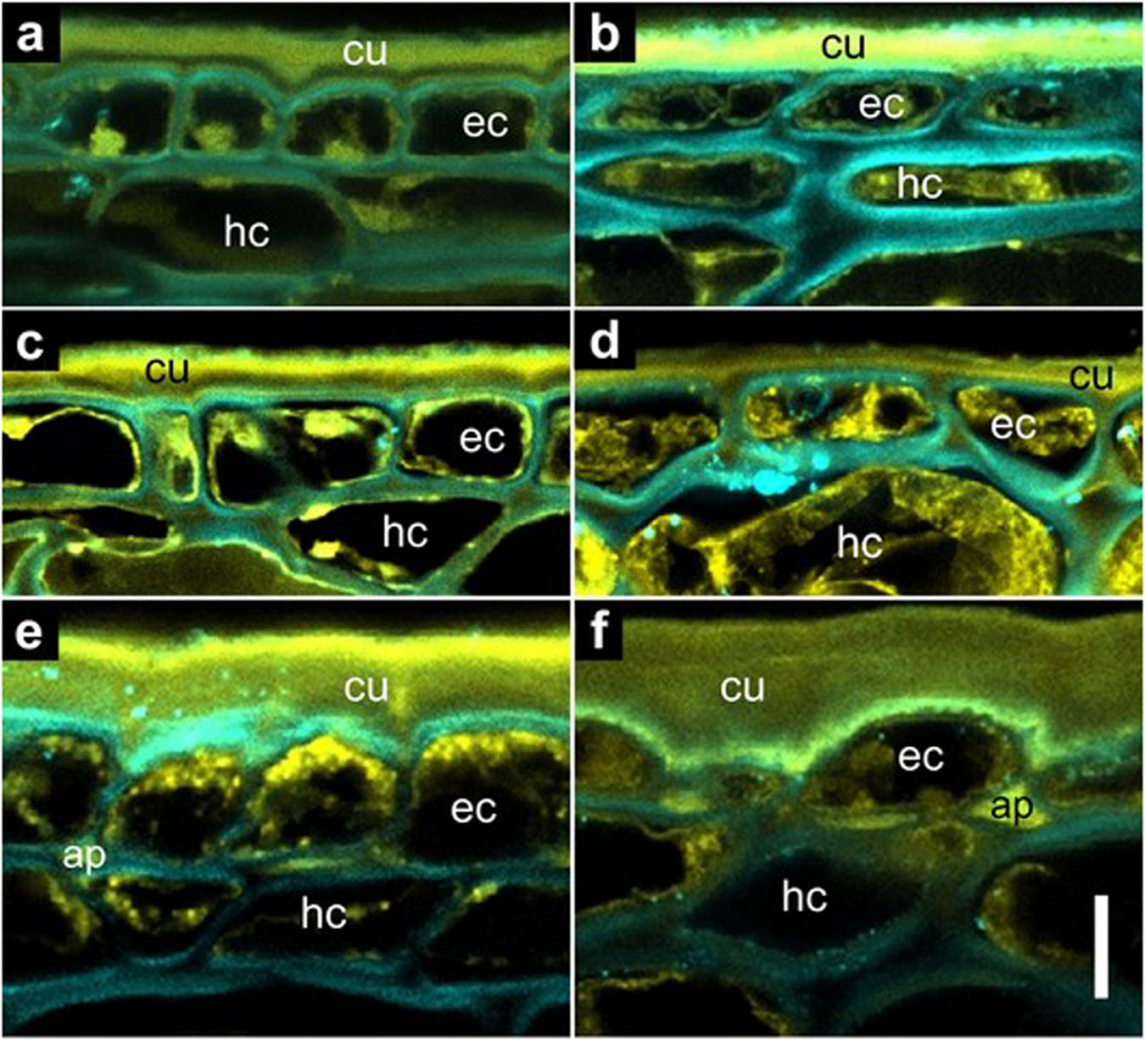
Confocal laser scanning micrographs of grape berry skin tissues. **a** ‘Merlot’ green hard berry. **b** ‘Merlot’ overripe berry. **c** ‘Zinfandel’ green hard berry. **d** ‘Zinfandel’ overripe berry. **e** ‘Concord’ green hard berry. **f** ‘Concord’ overripe berry. The autofluorescence (501–549 nm) from phenolic compounds was visualized by yellow false color. The fluorescence (422–464 nm) from cell walls due to the Calcofluor white staining of cellulose was visualized by blue false color. Overlapping blue and yellow signals are responsible for the greenish appearance of some structures. The letters indicate cuticle (cu), epidermal cells (ec), hypodermal cells (hc), and anticlinal pegs (ap). The vertical scale bar represents 10 µm

The morphology of berry skin tissues of three genetically diverse grape cultivars was visualized by confocal laser scanning micrography (Fig. 1). We compared green hard (GH) berries and overripe (OR) berries to determine whether any differences observed in mature berries were already present in immature berries, i.e., before the start of ripening. The cuticle of *Vitis vinifera* L. ‘Merlot’ and ‘Zinfandel’ berries formed only on the peripheral side of epidermal cells, whereas highly cuticularized anticlinal pegs protruded between or below some epidermal cells of OR berries of ‘Concord’, an interspecific hybrid with *Vitis labrusca* L. and *V. vinifera* ancestry. Similar cuticle thickness was observed in ‘Merlot’ berries at the GH and OR stages (*p* = 0.98), while the cuticle thickness decreased slightly (*p* < 0.05) in ‘Zinfandel’ and increased by 27% (*p* < 0.001) in ‘Concord’ from the GH to the OR stage. Irrespective of the developmental stage, ‘Concord’ berries had a two- to three-fold thicker cuticle than ‘Merlot’ and ‘Zinfandel’ berries (Table 1). The epidermal cells of GH berries showed square to rectangular shapes, and the interior epidermal cell walls were aligned in the same surface (Fig. 1a, c, e). By contrast with the GH stage, the epidermal cells were flattened or tangentially stretched at the OR stage in all three cultivars, and the inner cell walls of the epidermis in ‘Zinfandel’ had a wavy pattern (Fig. 1b, d, f). Some key differences in hypodermal cell traits were apparent despite the limited number of observed cell layers. Beneath the epidermal cells, the hypodermis of OR ‘Merlot’ berries was aligned in layers and flattened (Fig. 1b). However, the hypodermal cells of OR ‘Zinfandel’ and ‘Concord’ berries were of irregular shapes (Fig. 1d, f). The hypodermal cells of ‘Zinfandel’ berries, moreover, were much larger than those of the other two cultivars.

**Table 1:** Total soluble solids (TSS), radius (*r*), fresh weight (FW), cuticle thickness (*t*_*c*_), estimated critical shell tension (*T*_*cs*_), and estimated yield tension (*T*_*y*_) in green hard (GH) and overripe (OR) berries of ‘Merlot’, ‘Zinfandel’, and ‘Concord’ grapevines

Cultivar	Stage	TSS	*r*	FW		*t*_*c*_		*T*_*cs*_	*T*_*y*_
		(°Brix)	(mm)	(g)	n^x^	(µm)	n^y^	(N/m)	(N/m)
‘Merlot’	GH	8.8 ± 0.4 a^v^	4.03 ± 0.02	0.27 ± 0.00 b	3	3.57 ± 0.20 b	4	nd^z^	nd
	OR	26.9 ± 0.3 B^w^	5.82 ± 0.07 C	0.89 ± 0.02 C	10	3.58 ± 0.13 B	4	526.6	40.4
‘Zinfandel’	GH	7.3 ± 0.1 b	4.66 ± 0.23	0.43 ± 0.05 ab	7	3.88 ± 0.09 b	5	nd	nd
	OR	30.5 ± 0.7 A	7.47 ± 0.17 B	1.82 ± 0.13 B	10	3.10 ± 0.09 B	5	261.7	14.9
‘Concord’	GH	5.0 ± 0.2 c	4.83 ± 0.28	0.53 ± 0.07 a	3	7.26 ± 0.31 a	5	nd	nd
	OR	21.5 ± 0.2 C	8.53 ± 0.07 A	2.92 ± 0.07 A	10	9.21 ± 0.42 A	5	497.6	116.8

^v^Mean ± SE. Different lower-case letters within columns indicate significant varietal differences by multiple range comparison (LSD) in GH berries.

^w^Different upper-case letters within columns indicate significant varietal differences by multiple range comparison (LSD) in OR berries.

^x^Number of berries used for TSS, *r*, and FW measurements.

^y^Number of berries used for *t*_*c*_ measurements; *t*_*c*_ was measured on 3‒9 equatorial positions per berry and averaged.

^z^*T*_*cs*_ and *T*_*y*_ were not estimated for GH berries because they do not behave like thin-shell spheres^10^.

The increase in *r* between GH and OR berries was 44%, 60%, and 77% in ‘Merlot’, ‘Zinfandel’, and ‘Concord’, respectively, and the fresh weight (FW) of OR berries was 3.3, 4.3, and 5.6 times greater than the FW of GH berries (Table 1). If berries are perfect spheres, the increase in surface area from GH to OR was 109%, 157%, and 212% in OR ‘Merlot’, ‘Zinfandel’, and ‘Concord’ berries, respectively. Assuming a homogenous cuticle thickness over the entire berry, the estimated total volume of the cuticle was 2.1, 2.1, and 4.0 times higher in OR berries than in GH berries of ‘Merlot’, ‘Zinfandel’, and ‘Concord’.

A customized water injection test was used in conjunction with thin-shell theory to estimate how *P*_*i*_ translates to *σ*_*h*_ on the skin of mature berries sampled at the OR stage; this approach cannot be applied to GH berries because, unlike ripening berries, immature berries do not behave like thin-shell spheres^10^. The estimated critical shell tension (*T*_*cs*_) at the point of berry splitting in ‘Zinfandel’ was half that in ‘Merlot’ and ‘Concord’ (Table 1). The estimated yield tension (*T*_*y*_) at the transition from elastic to plastic deformation under pressure in ‘Merlot’ and ‘Concord’ was 2.7 and 7.8 times higher than the estimated *T*_*y*_ in ‘Zinfandel’.

**Fig. 2:**
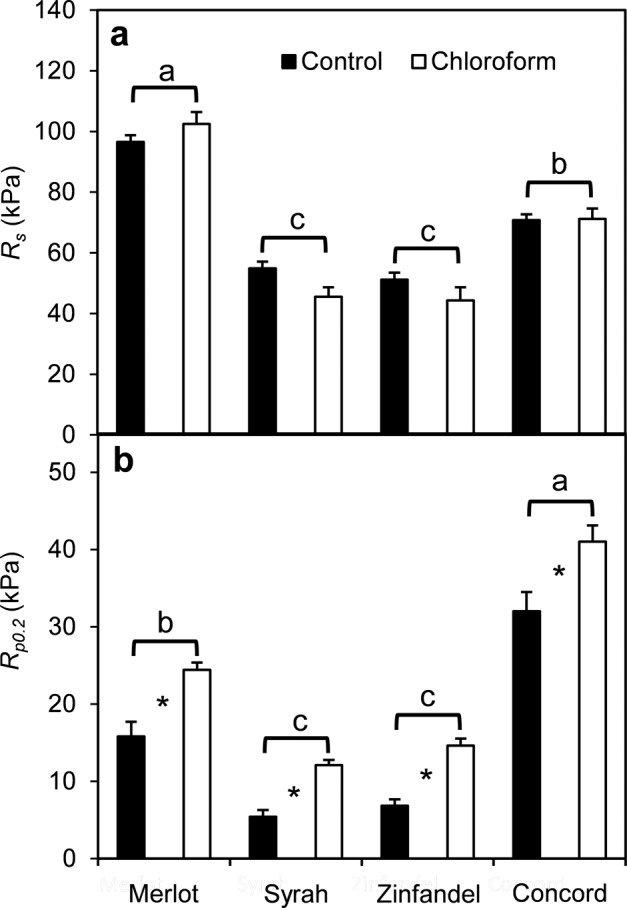
Effect of partial cuticular wax removal on mechanical properties of grape berries. **a** Splitting resistance (*R*_*s*_). **b** Offset yield strength (*R*_*p0.2*_). ‘Merlot’, ‘Syrah’, ‘Zinfandel’, and ‘Concord’ grape berries were immersed in chloroform for 20 s. The error bars indicate standard errors of the mean (*n* = 10 for ‘Merlot’, ‘Syrah’, ‘Zinfandel’; *n* = 5 for ‘Concord’). Asterisks indicate significant differences (*p* < 0.05) between control and chloroform treatment by Student’s *t*-test. Letters above brackets indicate significant differences among cultivars by Fisher’s least significant differences test

**Fig. 3:**
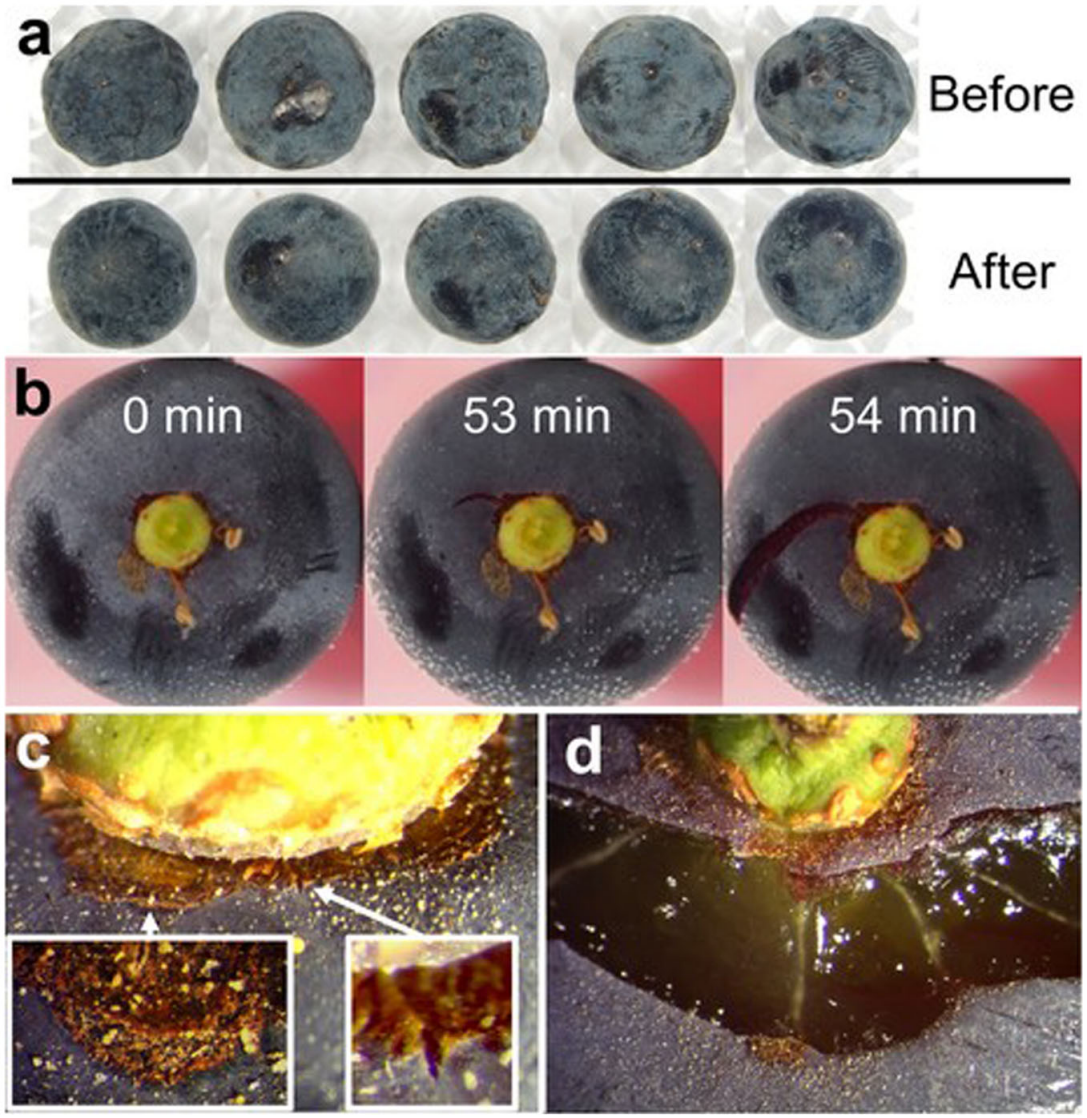
Grape berry surface observations. **a** Appearance of dehydrated ‘Merlot’ berries before and after immersion in chloroform for 20 s. **b** Temporal progression of splitting on ‘Concord’ berry immersed in water. The time after the start of berry submersion is noted in each panel. **c** Distribution of pre-existing microcracks on the receptacle area or floral cap scar on ‘Concord’ berry. The left inset and arrow indicate a concentric microcrack. The right inset and arrow indicate a radial microcrack. **d** A split propagating bilaterally from a concentric microcrack; the radial whitish traces in the exposed berry flesh are vascular bundles

The effects of partial cuticular wax removal by brief (20 s) immersion in chloroform on the rheological properties of mature ‘Merlot’, ‘Syrah’, ‘Zinfandel’, and ‘Concord’ berries sampled at the blue (B) and ripe (R) stages were mixed. The chloroform treatment did not alter the splitting resistance (*R*_*s*_ = *P*_*i*_ at splitting) of the berries (Fig. 2a) but increased the offset yield strength at 0.2% strain (*R*_*p0.2*_ = *P*_*i*_ at the transition from elastic to plastic deformation as defined by ASTM International) by 1.3-fold to 2.2-fold in all cultivars (Fig. 2b). When extra detached but otherwise intact (i.e., without wax removal) ‘Merlot’ berries were left to transpire for 9 days under standard laboratory conditions the berries lost 19% of their FW. The wrinkled appearance of the berry surface indicated significant dehydration (Fig. 3a). Subsequent immersion in chloroform for 20 s removed about 1.5 mg of cuticular waxes per berry and smoothed out most of the dehydration-induced wrinkles from the berry surface. However, some patches and lines of whitish epicuticular wax remained as a visible indication that the brief chloroform treatment did not extract all of the wax (Fig. 3a).

The initiation and propagation of splits on grape berries immersed in water were observed by time-lapse photography. Cuticular cracks originated at and extended from existing flaws or microcracks to fully developed splits that exposed the berry flesh (Fig. 3b). The temporal dynamics of propagation showed slow creep for an extended time, then rapid elongation over the last minute. The time required for a microcrack to develop into a fully extended split spanned from 25 to 227 min in the observed four samples. In this and all other berry immersion tests reported below, splitting was strictly associated with the region near the receptacle (Fig. 3c, d). The splits did not penetrate through the whole flesh layer but propagated widely across the berry surface. Pre-existing microcracks were associated with the area close to the receptacle or suberized scars where the calyptra (flower cap) had been attached before anthesis. These cap scars had five protruding bulges around the receptacle on the skin. Two types of microcracks were observed that were embedded in the highly suberized area (Fig. 3c). Radial microcracks initiated from the base of the berry near the receptacle. The other type of microcracks was on the edge of the cap scars and concentric with the pedicel. Depending on the splitting pattern, microcracks initiating from the berry-pedicel junction (Fig. 3b) could be differentiated from those initiating from the edge of a cap scar (Fig. 3d).

Because ‘Concord’ was the most splitting-susceptible cultivar in this study, survival times and hazard ratios of ‘Concord’ berries immersed in different aqueous solutions were analyzed by Kaplan–Meier (KM) survival curves and Cox Hazard Regression (CHR). The survival time of a berry is defined as the duration from the start of immersion to the moment of berry splitting. The hazard ratio is defined as the hazard rate of the treatment group divided by that of the control group, where the hazard rate is the probability that a berry splits at any moment during the experiment. In a first test, we evaluated the effect of solution pH on berry splitting, because protons are involved in cell wall loosening. Berries were sampled at the OR stage (TSS = 21.3 ± 0.3 °Brix; *n* = 21). The berries in pH 3.3 solution had a median survival time (defined as the time at which the ratio of intact, nonsplit berries to total berries became ≤ 0.5) of 128 min, while the berries in the pH 7.0 solution maintained a survival probability above 60% until the observation ended after 9 h (Fig. S1). In other words, nearly two-thirds of the berries at pH 7.0 did not split during the test, whereas only 10% of the berries at pH 3.3 remained intact. Moreover, the hazard ratio between the pH 3.3 and pH 7.0 solutions was 3.9 (*p* < 0.05), indicating that berries in the pH 3.3 solution had an almost fourfold greater chance to split than those in the pH 7.0 solution.

**Fig. 4:**
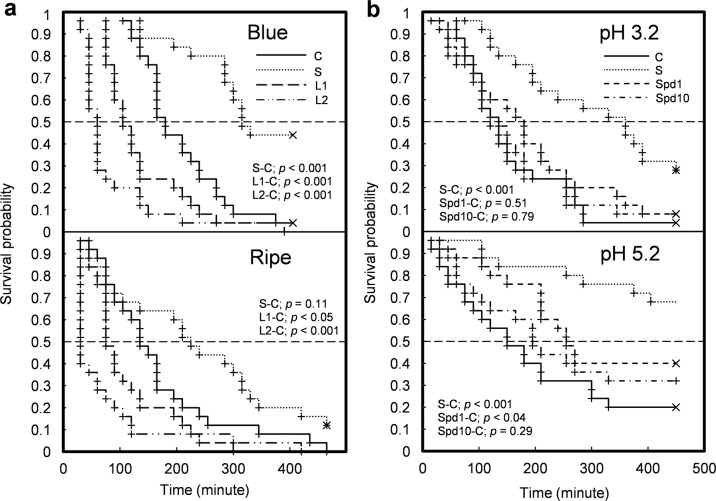
Survival probability of ‘Concord’ grape berries in H_2_O_2_-based immersion solutions. **a** Blue and ripe berries in control (C), stiffening (S), loosening 1 (L1), and loosening 2 (L2) solutions. All solutions were based on 50 mM sodium citrate buffer at pH 3.3. Control: buffer solution. Stiffening: 50 mM H_2_O_2_ added. Loosening 1 & 2: 50 mM H_2_O_2_ + 50 mM ascorbate added. Loosening 2 received 15 min pre-treatment in 1 mM FeSO_4_. All other treatments received 15 min pre-treatment in the buffer. **b** Overripe berries in control (C), stiffening (S), 1 mM spermidine (Spd1), and 10 mM spermidine (Spd10) solutions with 50 mM sodium citrate buffer at pH 3.2 and pH 5.2. The C group was the reference for comparisons using the Gehan–Breslow–Wilcoxon test. Significance is noted for comparison pairs; *p*-value. Plus signs indicate individual splitting events. Multiplication signs indicate censored events, i.e., intact berries at the end of the experiment

Next, to test the effect of H_2_O_2_-based cell-wall loosening and stiffening solutions at pH 3.3 (for solution composition see Table 2), ‘Concord’ berries were sampled at the B and R stages, respectively. The interval between sampling dates was long enough to permit the berries to accumulate sugar (*p* < 0.05; Table S1). Generally, B berries remained intact longer in cell-wall manipulation solutions than the more mature R berries. The KM survival curves showed that B berries did not split during the first hour except when immersed in the cell-wall loosening solution L2, which was pretreated with FeSO_4_ to enhance Fenton’s reaction (Fig. 4a). In contrast, the first split R berries in all treatments were found within the first hour. The median survival time of the B berries in cell-wall stiffening (S) solution was 315 min, whereas the median survival time of the R berries in L2 solution was only 30 min (Table 2). Compared with the control, the S treatment extended the survival time by 135 min in B berries (*p* < 0.001) but not in R berries (*p* = 0.11). On the other hand, the L1 solution (without FeSO_4_ pretreatment) and L2 solution shortened the survival time by 60 and 120 min in the B and R berries (*p* < 0.05). The CHR analysis indicated a hazard ratio of 1.1 for every 1 °Brix increment (*p* < 0.05), indicating that the splitting probability increased with increasing TSS of the ripening berries. The hazard ratio of L1 to C was 1.7 (*p* < 0.01) and the ratio of L2 to C was 2.9 (*p* < 0.001), while the ratio of S to C was 0.4 (*p* < 0.001). These results indicate that cell-wall loosening increased the chance of berry splitting, whereas cell-wall stiffening reduced berry splitting compared with the control.

**Table 2:** Median survival time (time at which ≤50% of the berries remained intact, i.e., nonsplit) of ‘Concord’ grape berries at blue and ripe developmental stages when the berries were immersed in cell wall manipulation solutions

	Blue		Ripe		Blue vs. Ripe
Treatment^x^	Time (min)	*p*	Time (min)	*p*	*p*
Control	180	*Ref*. ^y^	135	*Ref*.	<0.05
Stiffening	315	<0.001^z^	225	0.11	<0.01
Loosening 1	105	<0.001	75	<0.05	<0.1
Loosening 2	60	<0.001	30	<0.001	<0.05

^x^All treatment solutions were based on 50 mM sodium citrate buffer at pH 3.3. Control: buffer solution. Stiffening: 50 mM hydrogen peroxide (H_2_O_2_) added. Loosening 1 & 2: 50 mM H_2_O_2_ + 50 mM ascorbate added. Loosening 2 received 15 min pre-treatment in 1 mM FeSO_4_. The other treatments received 15 min pre-treatment in the buffer.

^y^The control for blue and ripe was used as the reference for comparisons within stages.

^z^Paired comparisons by Gehan–Breslow–Wilcoxon test.

Finally, the effects of Spd were compared to H_2_O_2_ application at pH 3.2 and pH 5.2, using ‘Concord’ berries sampled at the OR stage. Other than TSS (>20 °Brix), the berry characteristics were similar to those of berries collected at the R stage (Table S1). The CHR model showed that the hazard ratio of every 1 pH increment of the solution pH was 0.7 (*p* < 0.001), indicating that the berries had a lower chance to split as the solution pH increased. The hazard ratio between H_2_O_2_ treatment and control was 0.37 (*p* < 0.001), confirming that H_2_O_2_-induced cell-wall stiffening reduced the probability of berry splitting. The addition of Spd was not effective relative to the control, except the 1 mM Spd treatment (Spd1) in pH 5.2 solution (*p* < 0.05). Applications of H_2_O_2_ increased the median survival time by 225 and 300 min in solutions of pH 3.2 and pH 5.2, respectively, and the Spd1/pH 5.2 solution increased the median survival time by 205 min (Fig. 4b).

## Discussion

The present study demonstrated that the fruit cuticle and skin cell walls have both common and unique roles in grape berry splitting. Both cuticle and cell walls bear tensile stress in the berry skin and may contribute to the mechanical properties of the skin. While the cuticle is the barrier that minimizes water exchange, microcracks in the cuticle may function as sites for localized water movement and as stress concentrators that initiate split propagation. However, cuticle thickness was not sufficient to explain the varietal and developmental differences in estimated *T*_*cs*_ and *T*_*y*_ in ‘Merlot’, ‘Zinfandel’, and ‘Concord’ grape berries after berry softening. Despite their much thicker cuticle and the increase in cuticle thickness during fruit ripening, ‘Concord’ berries were much more susceptible to splitting than ‘Merlot’ berries whose thinner cuticle did not change during ripening (see also ref. ^10^). It is possible that other cuticular traits, such as chemical composition or density, might differ among these cultivars. For instance, ‘Concord’ berries have lower cuticular conductance and transpiration rates than ‘Merlot’ and ‘Syrah’ berries, which could be caused by differences in cuticle thickness or wax amounts and/or composition^34^. Also, our measurements of cuticle thickness could not determine the role, if any, of the anticlinal cuticular pegs between the epidermal cells in strengthening or weakening the skin of ‘Concord’ berries. Clearly, their thick cuticle and anticlinal pegs did not prevent ‘Concord’ from being the most splitting-susceptible grape cultivar in our study.

It remains to be determined whether the anticlinal pegs weaken or strengthen the continuum of the tensile-stress bearing epidermis after berry softening at the onset of ripening. It is also possible that their low transpiration rates^34^ contribute to the high splitting susceptibility of ‘Concord’ berries by rendering them less able to discharge surplus phloem water^8,9^, which could increase *P*_*i*_ under conditions of low vapor pressure deficit (VPD). Unlike in ‘Merlot’, ‘Concord’, and sweet cherries^21^, the cuticle of ‘Zinfandel’ grape berries apparently bears a higher proportion of *σ*_*h*_ due to the lower support from skin cell walls or anticlinal pegs. The decrease in cuticle thickness in ‘Zinfandel’ from the GH to OR stage may have been due to stretching by *σ*_*h*_^35^. While the epidermal cell walls in ripening ‘Merlot’ berries were deformed by *σ*_*h*_ acting on the walls, the stress might be transmitted to the cuticle in ‘Zinfandel’ berries whose epidermal cell walls assumed a wavy pattern, implying they were not under tensile stress. It seems likely that this is a genotype effect rather than an artifact of sample preparation because the ‘Merlot’ epidermis did not show this wavy pattern.

Using chloroform to partially remove cuticular wax did not change *R*_*s*_ but consistently increased *R*_*p0.2*_ of ripening grape berries, indicating that the chloroform treatment made the berries reluctant to deform plastically. This demonstrates that changes in the cuticular waxes alter the mechanical properties of the whole berry, even though the short-duration (20 s) chloroform dipping was likely to have extracted predominantly epicuticular wax, and then incompletely so. The recovery of smooth skin on dehydrated berries following chloroform treatment showed that partial wax removal reversed the normal process of strain fixation due to wax deposition^23^. Further research is required to determine how epi- and intracuticular waxes contribute to the mechanical properties of the skin. Nevertheless, while a higher *R*_*p0.2*_ implies a higher *P*_*i*_ is required to deform the skin plastically^10^, the inconsistent response of *R*_*s*_ to wax removal suggests that cuticular wax is not directly involved in the determination of skin fracture properties. On the other hand, wax removal would have decreased the safety margin, defined as the difference between *R*_*p0.2*_ and *R*_*s*_^10^, which suggests a higher splitting probability if berry expansion continues. Although it has been suggested that wax removal should decrease the stress that causes skin fracture^36^, those studies tested mechanical properties of isolated cuticle sections, which might have excluded microcracks on the sample surface.

The pre-existing microcracks near the receptacle area and cap scars of the berries used in our study would have provided potential access points for water absorption. These microcracks also work as stress concentrators as splitting is initiated. As the preferred infection site for *Botrytis cinerea* in grapes, these microcracks might form as early as anthesis when the flowers shed their protective calyptra or fused petals, leaving behind a “cap scar”^37,38^. Unlike in a pressurized vessel enclosing a flowing liquid, cell membranes prevent most of the internal contents of flesh cells from escaping when a berry splits, and the initiation of split propagation might involve bursting of only a few cells, likely as a result of localized osmotic water uptake^39^. Because the *σ*_*h*_ generated by *P*_*i*_ is not fully released until the total span of a split has been reached, the release of skin stress must occur by releasing skin strain or creating new surfaces by splitting but not, or only partially, by oozing cell sap. This stress-relief process also determines the size of a split. In our study, the splitting process started slowly, sometimes extending over hours until accelerating in the final propagation stage due to the rapid stress concentration effect of the increasing split size.

Manipulating the strength of skin cell walls, using ROS-related reactions and pH changes, altered the survivability of grape berries in immersion solutions: wall stiffening treatments delayed splitting and lowered the splitting probability, whereas wall loosening treatments accelerated splitting and increased the splitting probability. The pH of apoplastic sap in ‘Concord’ berries was found to change from about 3.5 to >4.5 during early ripening, and the sap’s buffering capability decreased due to the low organic acid concentration in the apoplast^40^. Therefore, the immersion solutions with pH 3.3 used here may have increased the proton concentration in the skin cell walls. While the berry skin is considered a hemicellulose-rich region^30^, the low apoplast pH would hinder hydrogen bonding with pectin. Alternatively, or additionally, an increase in expansin gene expression during berry ripening^41^ might also explain why skin cell walls were sensitive to low pH treatments. Adding both ascorbate and H_2_O_2_ demonstrated that ferrous ions in the POX of skin tissues of grape berries are sufficient to trigger Fenton’s reaction^29,33,42^. Without ascorbate, the H_2_O_2_ solution might trigger cross-linking of structural proteins (e.g. extensin) through POX, which was found to increase yield strength of primary cell walls in grape callus cultures^28,43^. Although the Spd treatment seemed to stiffen skin cell walls and reduced the probability of berry splitting in the high-pH solution, Spd was not as effective as exogenous H_2_O_2_, suggesting that polyamine oxidase may be inactive in the berry cell walls. As an antioxidant, Spd may have dissipated some H_2_O_2_, thus resulting in similar survival responses in the control and Spd10 treatment^33^. Future work examining enzyme activities and gene expression would help to elucidate the mechanisms involved in the modification of cell-wall strength.

Our results partially support both the traditional critical turgor model^16^ and the recently proposed zipper model^21,39^, suggesting that the two models of fruit splitting should be viewed as complementary rather than incompatible. In an earlier study we found that splitting resistance was unaltered by berry dehydration but declined markedly during berry softening at the onset of grape ripening^10^, i.e., after or concomitant with a decline in cell turgor^44^. Working with sweet cherries, Winkler et al. (ref. ^45^) argued that fruit splitting is independent of fruit turgor and should be regarded as a local phenomenon, because fruit partially immersed in water split even when the other side of the fruit was dehydrating, and because the water volume that caused splitting was higher in injection tests than in immersion tests^45^. We contend that such local phenomena should be interpreted as indicating that critical pressure may build up regionally in immersion tests or during rain events. Stiffening or loosening of skin cell walls in our study was enough to decrease or increase, respectively, the probability of splitting when grape berries were immersed in different aqueous solutions. The action of our immersion solutions may have been localized to microcracks because hydrophobic waxes may prevent interaction between cell walls and reagents. However, splitting also occurred in the absence of liquid surface water on ripening berries of splitting-susceptible grape cultivars whose root system was pressurized, as well as during humid nights or when berry transpiration and/or xylem backflow were restricted^8,9,17^. Under high humidity (low VPD) and continued phloem inflow, water may accumulate in a berry and increase *P*_*i*_ due to reduced transpiration^9,34^. Generally, internal pressure in fruit is determined by the fruit water balance, and *P*_*i*_ can reach the critical turgor level globally by phloem inflow alone^8,17^ or regionally by localized surface water absorption^45^.

In conclusion, by integrating morphological and anatomical observations with principles of thin-shell theory and fracture mechanics, as well as combining biochemical manipulations with survival analysis, this study provides evidence for the idea that the cuticle and the skin cell walls together bear the tensile stress transmitted from internal tissues to the skin of ripening grape berries. The turgor pressure of flesh cells not only drives the expansive growth of a berry but also generates tensile stress in the cuticle and skin cell walls during ripening. Berry splitting occurs when these external structures fail to dissipate the stress and concentrate it to an existing flaw, termed microcrack, in the cuticle. Cell wall manipulations demonstrated that the skin cell walls bear tensile stress in the skin, and ROS alter skin strength in a localized fashion acting on or near microcracks. Consequently, cuticular cracks originated at and extended slowly from microcracks over an extended time and then suddenly widened to fully developed splits that exposed the berry flesh. However, we were unable to estimate localized stress in the skin due to the complex (i.e., nonhomogenous) structure of the cuticle and cell walls. Therefore, the relative contributions of the cuticle and skin cell walls to the stress-bearing capacity of the skin remain to be determined.

## Materials and methods

### Plant material and berry sampling

For measurements of cuticle thickness and effects of cuticular wax removal, own-rooted grapevines ‘Merlot’, ‘Syrah’, and ‘Zinfandel’, as well as ‘Concord’ were selected from the vineyard at the Roza experimental farm (46°17’18” N; 119°43’56” W; elevation 345 m) of the Irrigated Agriculture Research and Extension Center near Prosser, Washington, USA, in 2015. For skin cell wall manipulations, own-rooted ‘Concord’ grapevines were selected from the vineyard at the center’s headquarter unit (46°15’10” N; 119°44’02” W; elevation 256 m) in 2015 and 2016. Both vineyards were drip-irrigated. Grape berries were sampled and classified into well-defined maturity groups or developmental stages named green hard (GH), green soft (GS), blush/pink (BP), red/purple (RP), blue (B), ripe (R), and overripe (OR)^46^. This stratified sampling method minimized the variation introduced by the asynchronous ripening of berries on the same or different fruit clusters.

### Cuticle thickness

To measure the thickness of grape berry cuticles, the confocal laser scanning microscopy method was adopted and modified^47^. During method optimization, we found no obvious differences in the outer edge of the cuticle between images observed by this approach and by bright field microscopy, and our measurements agreed well with cuticle thickness measurements acquired using bright field microscopy^48^. ‘Merlot’, ‘Zinfandel’, and ‘Concord’ grape berries were sampled at GH (pea-size; diameter > 7 mm) and OR stages. The berry *r* and FW were measured. A skin sample was prepared from each berry by cutting a cube with sides of 3 mm from the equatorial area of the berry and immediately fixing it in formalin-acetic acid [5% formalin (37% formaldehyde, aqueous), 5% glacial acetic acid, 45% ethanol, 45% distilled water (v/v)]. The TSS concentration in juice expressed from the remaining berry flesh was measured by refractometry (Quick-Brix 60, Mettler-Toledo, Schwerzenbach, Switzerland). Cryoprotection of the skin samples was achieved by successive replacement of sucrose solutions until the sucrose concentration reached 20% (w/w). The samples were embedded in optimum cutting temperature medium (Tissue-Tek, Sakura, Nagano, Japan) and stored at −20 °C. Cryosections of 30 µm were prepared using a cryomicrotome (Cryocut 1800, Leica, Nussloch, Germany). The specimens were collected and floated on Calcofluor white (0.1% w/v in distilled water) staining solution for 2 min. By staining crystalline cellulose, Calcofluor white is useful to delineate the cuticle/cell-wall boundary^47^. Preliminary tests using the lipid fluorescent stain auramine O indicated no improvement for cuticle observation and no change in apparent cuticle thickness; thus, no further staining was applied. After staining, the specimens were rinsed with water and sealed in water on slides with nail polish under a coverslip. The slides were stored at 1 °C until microscopy examination. Images were generated using a Leica TCS-SP8 Confocal Laser Scanning Microscope (Leica Microsystems, Wetzlar, Germany). The emissions of phenolics and Calcofluor white were observed at 501–549 nm and 422–464 nm, respectively, by excitation at 405 nm with a near-UV diode. The cuticle was visualized by phenolic autofluorescence signals with yellow false color, and cell walls were visualized by fluorescence signals resulting from Calcofluor white staining of cellulose with blue false color. LAS-X software (Leica Microsystems) was used to determine the thickness of the cuticle, defined as the shortest distance from the edge of the outer epidermal cell walls to the outermost surface of the cuticle, and calculated from the average of three to nine measurements in each skin sample.

### Skin physical properties and wax removal

Varietal differences and changes in cuticle thickness between the R and OR stages of ‘Merlot’, ‘Zinfandel’, and ‘Concord’ grape berries were compared to varietal differences and developmental changes in berry *r* and *R*_*s*_. The latter was quantified using the berry injection test described elsewhere^10^. Briefly, pressurized water was injected gradually into the locular space of a berry, and changes in berry *r* and *P*_*i*_ were monitored until the berry split. Critical shell tension (*T*_*cs*_) at the point of splitting and offset yield strength at 0.2% strain (*R*_*p0.2*_) provided in Table S2 were estimated using regression relationships obtained in the previous study^10^. The tension at the start of an irreversible expansion, termed yield tension, was calculated as *T*_*y*_ = *R*_*p0.2*_ × *r*/2. In addition, cuticles were manipulated by partial removal of cuticular wax from ‘Merlot’, ‘Syrah’, ‘Zinfandel’, and ‘Concord’ berries. The effect of chloroform treatment on *R*_*s*_ and *R*_*p0.2*_ was evaluated in the injection test^10^. In the treated berries, before installing the berry-needle assembly on the adapter, the stylar half of the berry was immersed and shaken gently in chloroform for 20 sec to partially remove wax from the cuticle^49^. Five additional ‘Merlot’ berries were left on the laboratory bench at approximately 22 °C/40% RH for 9 days, at which time they showed visible signs of dehydration, before they too were immersed in chloroform for 20 s. The weight of the berries and the visual appearance of the berry surface before and after chloroform immersion was recorded.

### Splitting initiation and progression

Pre-existing cracks on the berry skin were examined by stereo microscopy (SteREO Discovery.V12, Carl Zeiss, Göttingen, Germany) and photographed by a digital camera (AxioCam ERc 5 s, Carl Zeiss). ‘Concord’ berries with their unsealed receptacles were fully immersed in deionized water to induce berry splitting, and the splitting process was recorded by time-lapse photography. Time-lapse frames were taken by a camera (E-M5; Olympus, Shinjuku, Japan) with a shutter release timer (AP-TR3L; Aputure, Shenzhen, China) at a speed of two frames per minute. The observation was ended once the split had fully developed.

### Skin cell wall manipulation

Immersion experiments to manipulate skin cell walls were carried out with ‘Concord’ grape berries. Preliminary trials indicated that the time to splitting in the immersion solutions of berries stored at 1 °C and 100% RH for up to 48 h did not differ from that of berries processed immediately after sampling, although storage increased the compressive strain (*ɛ*_c_) by 15% (*p* < 0.001) and decreased berry FW by 0.6% (*p* < 0.001). Therefore, berries that were sampled in the morning were held at 1 °C and 100% RH until they were processed on the same day. In 2015, the effect of immersion solution pH (50 mM sodium citrate buffer at pH 3.3 vs. pH 7.0) was tested in OR berries. The pH 7.0 solution served as the control. One hundred berries were collected in the vineyard and divided into five groups. The pedicels were carefully removed with a razor blade or scalpel. Berry maturity was estimated by measuring TSS in one designated group. Berry FW, *r*, and *ɛ*_c_ were measured. The *ɛ*_c_, which quantifies the magnitude of deformation due to a compressive force, was estimated using the skinfold caliper method as described previously^10^.

In 2016, 130 berries each at the B, R, and OR stages were collected, and 30 berries were used to measure FW, TSS, *r*, and berry elastic modulus (*E*_*b*_). The *E*_*b*_ was estimated using a customized device testing deformation and force while a berry is under compression as described elsewhere^46^. The remaining berries were divided into groups of 25 berries per treatment, and each berry was considered an experimental unit. The berries were immersed in solutions designed to manipulate cell wall properties. We used H_2_O_2_-related reactions^50^ to induce skin cell wall loosening or hardening in B and R berries, and we further tested the use of Spd to stimulate cell wall stiffening^33,50^ in OR berries. The H_2_O_2_ treatments included a control (C) solution of 50 mM sodium citrate buffer at pH 3.3; a stiffening (S) solution of 50 mM H_2_O_2_ in the buffer; two loosening solutions (L1 and L2) of 50 mM H_2_O_2_ + 50 mM ascorbate in the buffer. The berries for C, S, and L1 treatments were pretreated with buffer solution for 15 min, while the berries in the L2 treatment were pretreated with 1 mM FeSO_4_ in the buffer to enhance Fenton’s reaction. The stiffening/loosening treatments are listed in Table S3. A two-factor design was used for the Spd experiment to vary both the solution pH and Spd concentration. Berries were immersed in citrate buffer solutions with pH 3.2 or pH 5.2, and in 1 mM Spd or 10 mM Spd (Spd10). The Spd treatments are listed in Table S4.

During all immersion experiments, berries were checked for splitting every 15 min. Split berries were removed from the solution immediately after the examination. At the end of each experiment, all nonsplit berries were noted as “censored”. Berries that split due to physical damage inflicted during sample preparation were noted as “censored” as well. Survival analysis was conducted with Statistica 7 software (Palo Alto, California, USA). Treatment effects on berry survival times, defined as the duration from the start of immersion to berry splitting or to the end of the experiment, were analyzed by Kaplan–Meier (KM) survival curves. Median survival time was defined as the time at which half of the berries remained intact (nonsplit) in the solution. Cox Hazard Regression (CHR) was used to analyze the splitting probability and to calculate the hazard ratio between a treatment and the control.

## Supplementary information

Supplemental materials
