# Supplementary material for: Cuticle and skin cell walls have common and unique roles in grape berry splitting
Source: Hortic Res. 2021 Aug 1;8:168. doi: 10.1038/s41438-021-00602-2 (PMC8325674; doi:10.1038/s41438-021-00602-2)
Supplement: Supplementary file 1 — Supplemental materials [file 41438_2021_602_MOESM1_ESM.docx]

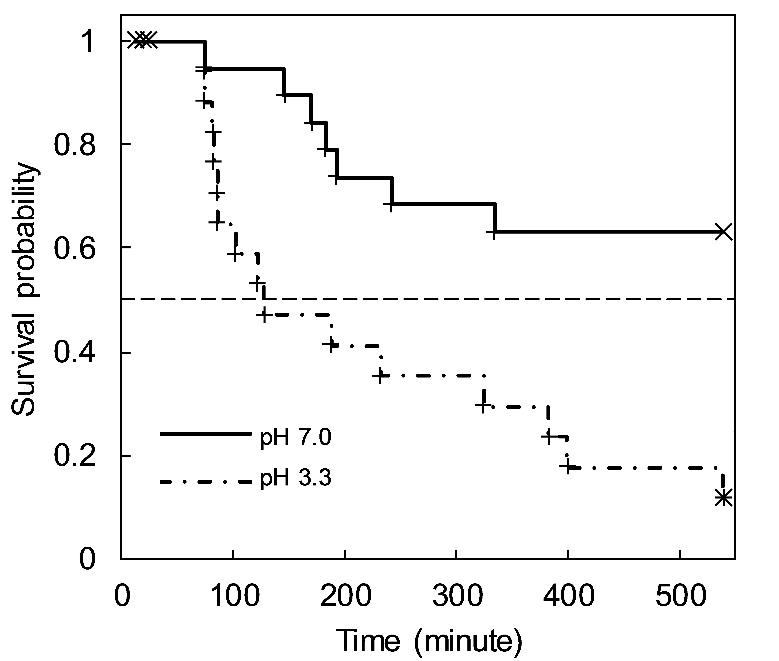


**Fig. S1 Survival probability of Concord grape berries in immersion test.** The ripe Concord grape berries (21.3 °Brix) were immersed in 50-mM sodium citrate buffer at pH 7.0 (solid line) or pH 3.3 (dash-dot line). The plus signs indicate the splitting events. The multiplication signs indicate censored events, including intact berries at the end of trial or damaged berries due to experiment preparation.

**Table S1** **Developmental stage, fresh weight (FW), total soluble solids (TSS), radius (*r*), and elastic modulus (*E_b_*) of Concord grape berries from three sampling dates in 2016.**

| Sampling date^x^ | Stage | FW  (g) | TSS  (°Brix) | *r*  (mm) | *E_b_*  (MPa) |
| --- | --- | --- | --- | --- | --- |
| 25 Aug | Blue | 2.31±0.07 b | 15.2±0.2 c | 7.97±0.08 b | 0.72±0.02 a |
| 21 Sep | Ripe | 2.53±0.08 a | 19.3±0.2 b | 8.25±0.08 a | 0.41±0.01 b |
| 3 Oct | Overripe | 2.46±0.07 ab | 20.5±0.2 a | 8.11±0.08 ab | 0.43±0.01 b |
| ^x^ Sample sizes were *n* = 30, 30, and 40 for 25 Aug, 21 Sep, and 3 Oct, respectively.  ^y^ Letters within columns indicate significant differences by Fisher’s LSD test (p < 0.05). | | | | | |

**Table S2** **Functions for the relationship between total soluble solids (TSS) and offset yield strength (*R_p0.2_*) and critical shell tension (*T_cs_*) in berries of three grape cultivars.**

| Cultivar | *R_p0.2_* (kPa) = *f*(TSS)^w,x^ | *T_cs_* (N/m) = *f*(TSS)^w,y^ | TSS range (°Brix) |
| --- | --- | --- | --- |
| ‘Merlot’ | −1.19x + 45.9 | –22.6x + 1134.5 | 9.5 – 25.3 |
| ‘Zinfandel’ | −0.61x + 22.6 | –18.6x + 829.0 | 10.1 – 29.0 |
| ‘Concord’ | 27.4^z^ | –42.7x + 1415.6 | 9.8 – 22.2 |
| ^w^ Regression analysis was based on previous results^10^.  ^x^ Functions predicting *R_p0.2_* at a given TSS (x).  ^y^ Functions predicting *T_cs_* at a given TSS (x).  ^z^ Because the correlation in Concord was not significant, the *R_p0.2_* at the ripe stage was used. | | | |

**Table S3** **Active agents in the immersion solution treatments used for grape berry skin cell wall manipulation**.

| Treatment | 15 min  Pre-incubation | Incubation |
| --- | --- | --- |
| Control (C) | Buffer only^x^ | Buffer only |
| Stiffening (S) | Buffer only | H_2_O_2_ (50 mM) |
| Loosening 1 (L1) | Buffer only | H_2_O_2_ (50 mM) + Asc^y^ (50 mM) |
| Loosening 2 (L2) | FeSO_4_ (1 mM) | H_2_O_2_ (50 mM) + Asc (50 mM) |
| ^x^ All agents were in sodium citrate buffer (50 mM) at pH 3.3.  ^y^ Ascorbate (Asc) | | |

**Table S4 Active agents in the immersion solution treatments used for grape berry skin cell wall stiffening at pH 3.2 or 5.2.**

| Treatments | pH^x^ | 15 min  Pre-incubation | Incubation |
| --- | --- | --- | --- |
| Control (C) | 3.2  5.2 | Buffer only  Buffer only | Buffer only  Buffer only |
| Stiffening (S) | 3.2  5.2 | Buffer only  Buffer only | H_2_O_2_ (50 mM)  H_2_O_2_ (50 mM) |
| Spermidine 1 mM (Spd1) | 3.2  5.2 | Buffer only  Buffer only | Spermidine (1 mM)  Spermidine (1 mM) |
| Spermidine 10 mM (Spd10) | 3.2  5.2 | Buffer only  Buffer only | Spermidine (10 mM)  Spermidine (10 mM) |
| ^x^All agents were in sodium citrate buffer (50 mM) at pH 3.2 or 5.2. | | | |
